# Attitudes, beliefs, and dependence related to electronic cigarettes among medical students at an eastern Anatolian university: a cross-sectional study

**DOI:** 10.3389/fpubh.2026.1934215

**Published:** 2026-09-24

**Authors:** Hülya Doğan Tiryaki, Sinem Çetinkaya, Melek Bilikli, Kevser Erdoğan, Osman Kurt

**Affiliations:** 1 Department of Public Health, Faculty of Medicine, Gaziantep University, Gaziantep, Türkiye; 2 Department of Public Health, Faculty of Medicine, İnönü University, Malatya, Türkiye; 3 Department of Public Health, Antalya Training and Research Hospital, Antalya, Türkiye

**Keywords:** attitudes, dependence, electronic cigarettes, medical students, public health, vaping

## Abstract

E-cigarette use has risen among young adults, and the attitudes, beliefs, and dependence of future physicians are relevant both to their own health and to their prospective role in patient counselling. This cross-sectional study assessed e-cigarette-related attitudes and beliefs, dependence among current users, and their correlates among medical students at the İnönü University Faculty of Medicine (Malatya, Türkiye) during 2025–2026. Using term-stratified random sampling, 366 students were analysed. The 18-item E-cigarette Attitude and Belief Scale in Adolescents (ECABA) was administered to all participants and the 22-item E-cigarette Dependence Scale (EDS-22) to current users. Data were analysed with non-parametric tests, Spearman correlation, and multivariable logistic regression. Ever and current e-cigarette use were 32.8 and 17.5%, respectively. ECABA scores increased significantly across non-users, occasional users, and regular users (medians 33, 39, and 48; *p* < 0.001). Male sex [adjusted odds ratio (aOR) 2.45, 95% CI 1.27–4.75] and a higher ECABA score (aOR 2.72 per 10 points, 95% CI 2.08–3.54) independently predicted current use, whereas age, cigarette smoking, and household smoking did not. Among current users, ECABA correlated with EDS-22 dependence (*r* = 0.561, *p* < 0.001). Pro–e-cigarette attitudes and beliefs were thus the strongest correlate of both use and dependence among medical students. Because these attitudes are modifiable, integrating structured, e-cigarette-specific education into medical curricula—particularly in the preclinical years—may help shape healthier attitudes in future physicians and strengthen their future role in tobacco counselling.

## 1 Introduction

Electronic cigarettes (e-cigarettes) have become one of the most rapidly diffusing nicotine products worldwide and are now regarded as a substantial public-health concern, particularly among adolescents and young adults (1). A recent systematic review and meta-analysis reported a global e-cigarette use prevalence of 22.7%, with the highest rates among males and in high-income settings (2). In Türkiye, where the sale and importation of e-cigarettes and their liquids are prohibited, these products nonetheless remain accessible through informal channels, and use among young people has continued to rise (3, 4).

Concern about e-cigarettes is driven both by their direct health effects and by their potential to promote a transition to combustible tobacco. An umbrella review of 56 prior reviews concluded that young people who vape face a consistent range of physical and psychological harms and are at higher risk of subsequently initiating cigarette smoking (1). Although the causal interpretation of this association remains debated between the gateway and common-liability frameworks (5, 6), mechanistic evidence indicates that early nicotine exposure can prime the young brain for dependence irrespective of the delivery route (7, 8).

Beyond behaviour itself, the cognitions that underlie e-cigarette use—attitudes and beliefs—are central to explaining and predicting uptake. Established behavioural frameworks, including the Theory of Planned Behaviour (9) and the Health Belief Model (10), hold that beliefs about a product’s risks and social value shape the intention to use it. Misperceptions that e-cigarettes are harmless or markedly safer than cigarettes have repeatedly been associated with greater use among university students (11, 12). Capturing these cognitions therefore requires validated, product-specific instruments; the E-cigarette Attitude and Belief Scale in Adolescents (ECABA) was recently developed and validated for this purpose (13).

Medical students constitute a group of particular interest. As future physicians they are expected to counsel patients on tobacco cessation and to act as role models, yet their own attitudes toward e-cigarettes are frequently understudied and their formal education on vaping is often limited (14). Studies of medical trainees in Türkiye and elsewhere have documented non-negligible use alongside important gaps in knowledge and preparedness (4, 15). Whether pro–e-cigarette attitudes translate into measurable dependence in this population, and how strongly attitudes predict use relative to conventional risk factors, has rarely been examined jointly with validated attitude and dependence measures. To our knowledge, no previous study has combined a validated e-cigarette-specific attitude scale with a validated dependence scale in medical students; consequently, the joint contribution of these modifiable cognitions to both use and dependence remains unquantified—the gap the present study was designed to address.

Accordingly, the present study aimed to (i) determine the prevalence of e-cigarette experimentation and current use among medical students at an eastern Anatolian faculty; (ii) quantify their e-cigarette-related attitudes and beliefs with the ECABA; (iii) assess dependence among current users with the EDS-22; and (iv) identify the independent correlates of current use, with particular attention to the contribution of attitudes and beliefs. We hypothesized that pro–e-cigarette attitudes and beliefs would be the strongest independent correlate of current e-cigarette use—over and above conventional risk factors—and would be positively associated with dependence among current users.

## 2 Materials and methods

### 2.1 Study design and setting

This descriptive, cross-sectional study was carried out at the İnönü University Faculty of Medicine, Malatya, Türkiye, during the 2025–2026 academic year. The manuscript was prepared in accordance with the STROBE reporting guideline for cross-sectional studies; the completed checklist is provided as Supplementary material.

### 2.2 Population, sampling, and participants

The source population comprised all 1,833 students enrolled across the six academic terms of the faculty. The minimum sample size was calculated with OpenEpi (version 3), assuming an expected prevalence of 50, 5% absolute precision, and 95% confidence, giving 318 participants; this figure was increased by 15% for non-response, for a target of 366. A term-stratified random sampling design was applied, with each term (1–6) treated as a stratum and students selected in proportion to stratum size using computer-generated random numbers. The questionnaire was distributed through a secure online link to 430 students (the target sample together with same-stratum reserves); 379 responses were received (response rate, 88.1%), of which 13 were excluded due to incomplete data, yielding 366 analysed participants with no missing data (Figure 1).

**Figure 1:**
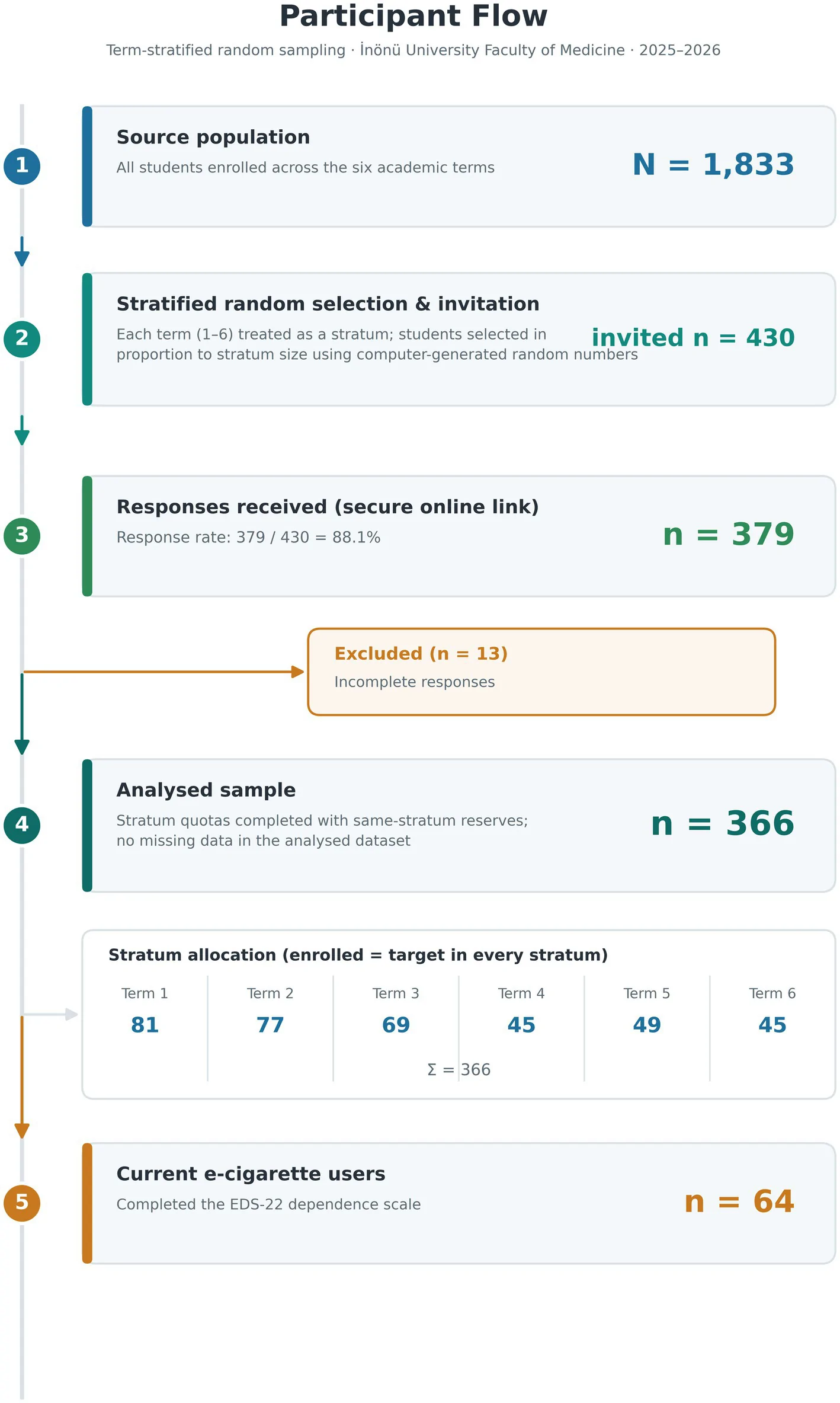
Participant flow diagram: source population, term-stratified proportional allocation, invitation of 430 students, responses received (response rate 88.1%), exclusion of incomplete responses, and the analysed sample.

### 2.3 Data collection instruments

Data were collected with a four-part self-report questionnaire recording sociodemographic characteristics; cigarette and e-cigarette behaviour (experimentation, current use and frequency, age at first use, and main source of access); and two validated scales.

The E-cigarette Attitude and Belief Scale in Adolescents (ECABA), developed and validated by Oksay et al. (13), comprises 18 items rated on a five-point Likert scale across five subscales (physical consequences; e-cigarettes versus packed cigarettes; establishing identification; e-cigarette addiction; socialization), yielding a total of 18–90, with higher scores indicating a stronger tendency toward e-cigarette use and weaker awareness of its harms.

The 22-item E-cigarette Dependence Scale (EDS-22), developed by Morean et al. (16) and validated in Turkish by Aslan and Onal (17), is rated from 0 to 4 and averaged to a score of 0–4, with higher values indicating greater dependence; it was completed only by current e-cigarette users.

### 2.4 Statistical analysis

Analyses were performed with IBM SPSS Statistics version 22.0 (IBM Corp., Armonk, NY, United States). Categorical variables are presented as counts and percentages and continuous variables as mean ± standard deviation and median (25th–75th percentile). Normality was assessed with the Kolmogorov–Smirnov test; because scale scores and age deviated from normality, non-parametric methods were used. Categorical comparisons used the Pearson chi-square test; continuous variables were compared with the Mann–Whitney U and Kruskal–Wallis tests, with Dunn tests and Bonferroni correction for pairwise comparisons. Spearman correlation was used to examine associations between continuous variables. Internal consistency was assessed with both Cronbach’s alpha (α) and McDonald’s omega (ω). Effect sizes are reported alongside *p*-values: Cramér’s V for chi-square tests, the rank-biserial correlation (r) for Mann–Whitney U tests, and epsilon-squared (ε^2^) for Kruskal–Wallis tests. Multicollinearity among the predictors in the logistic regression model was assessed with variance inflation factors (VIF). A multivariable binary logistic regression model was fitted with current e-cigarette use as the outcome and sex, age, current cigarette smoking, household smoker, and ECABA total score as predictors; calibration was assessed with the Hosmer–Lemeshow test. Statistical significance was set at *p* < 0.05.

### 2.5 Ethical considerations

The study was approved by the İnönü University Health Sciences Non-Interventional Research Ethics Committee (18 May 2026; decision no. 2026/10–256), and institutional permission was granted by the Deanery of the Faculty of Medicine (14 May 2026). The study followed the Declaration of Helsinki; participation was voluntary and anonymous, and informed consent was obtained from all participants.

## 3 Results

### 3.1 Participant characteristics

Of the 366 participants, 186 (50.8%) were male, and the median age was 22 years (IQR 20–24). Current e-cigarette use differed by sex, being more frequent among males (67.2% of current users) than females (*p* = 0.006, Cramér’s V = 0.144), and across academic terms (*p* = 0.027, Cramér’s V = 0.186); perceived income and household smoking did not differ between users and non-users (Table 1).

**Table 1:** Sociodemographic characteristics by current e-cigarette use.

Characteristic	Category	Total (*n* = 366)	Non-user (*n* = 302)	User (*n* = 64)	*p*
Age (years), median (IQR)		22 (20–24)	22 (20–24)	21 (20–24)	0.746
Gender, *n* (%)	Male	186 (50.8)	143 (47.4)	43 (67.2)	**0.006**
	Female	180 (49.2)	159 (52.6)	21 (32.8)	
Term, *n* (%)	1st	81 (22.1)	67 (22.2)	14 (21.9)	**0.027**
	2nd	77 (21.0)	62 (20.5)	15 (23.4)	
	3rd	69 (18.9)	50 (16.6)	19 (29.7)	
	4th	45 (12.3)	40 (13.2)	5 (7.8)	
	5th	49 (13.4)	47 (15.6)	2 (3.1)	
	6th	45 (12.3)	36 (11.9)	9 (14.1)	
Perceived income, *n* (%)	Low	40 (10.9)	34 (11.3)	6 (9.4)	0.697
	Middle	289 (79.0)	236 (78.1)	53 (82.8)	
	High	37 (10.1)	32 (10.6)	5 (7.8)	
Household smoker, *n* (%)	Yes	210 (57.4)	168 (55.6)	42 (65.6)	0.184
	No	156 (42.6)	134 (44.4)	22 (34.4)	

Chi-square test for categorical variables; Mann–Whitney U test for age. Bold *p*-values are statistically significant (*p* < 0.05). IQR, interquartile range.

### 3.2 Cigarette and E-cigarette use

Ever and current cigarette use were 53.3 and 29.8% (occasional plus regular), respectively. In total, 120 students (32.8%) had ever tried an e-cigarette and 64 (17.5%) were current users (Table 2). Among those who had tried e-cigarettes, the mean age at first use was 20.6 ± 2.7 years, and friends were the most frequently reported source of access (Table 2).

**Table 2:** Cigarette and e-cigarette use characteristics.

Variable	Category	*n* (%)
Ever tried cigarette	Yes	195 (53.3)
	No	171 (46.7)
Current cigarette use	None	257 (70.2)
	Occasional	36 (9.8)
	Regular	73 (19.9)
Ever tried e-cigarette	Yes	120 (32.8)
	No	246 (67.2)
Current e-cigarette use	None	302 (82.5)
	Occasional	11 (3.0)
	Regular	53 (14.5)
Frequency (current users, *n* = 64)	Daily	40
	Several days/week	14
	Rarely	5
	A few times/month	3
	Not reported	2
Main access source	Friends	83
	Social media	23
	Internet sites	3

Age at first e-cigarette use among those who had ever tried (*n* = 116): 20.6 ± 2.7 years (median 21).

### 3.3 E-cigarette use and attitudes by educational phase

When students were grouped by educational phase, current e-cigarette use was more frequent among preclinical (terms 1–3) than clinical (terms 4–6) students (21.1% vs. 11.5%, *p* = 0.027, Cramér’s V = 0.116), whereas ECABA attitude scores did not differ by phase (medians 36 vs. 34, *p* = 0.315, rank-biserial *r* = 0.062) (Table 3).

**Table 3:** E-cigarette use, cigarette use, and attitudes by educational phase.

Variable	Preclinical (*n* = 227)	Clinical (*n* = 139)	*p*
Current e-cigarette use, *n* (%)	48 (21.1)	16 (11.5)	**0.027**
Ever e-cigarette use, *n* (%)	81 (35.7)	39 (28.1)	0.164
Current cigarette use, *n* (%)	60 (26.4)	49 (35.3)	0.094
ECABA total, median (IQR)	36 (24–44)	34 (23–44)	0.315

Preclinical: terms 1–3; clinical: terms 4–6. Chi-square test (categorical) and Mann–Whitney U test (ECABA). Bold *p*-values are significant. ECABA, E-cigarette Attitude and Belief Scale in Adolescents; IQR, interquartile range.

### 3.4 Reliability and distribution of scale scores

The ECABA total score had a median of 35 (IQR 24–44) and excellent internal consistency (α = 0.921, ω = 0.924); subscale alphas ranged from 0.501 (socialization) to 0.940 (e-cigarettes versus packed cigarettes), with McDonald’s ω ranging from 0.525 to 0.942 (Table 4). Among current users, the EDS-22 had a median of 2.00 and very high reliability (α = 0.985, ω = 0.988) (Table 4).

**Table 4:** Reliability and distribution of ECABA (*n* = 366) and EDS-22 (current users, *n* = 64) scores.

Scale/subscale	Items	Range	Mean ± SD	Median (IQR)	Cronbach α	McDonald ω
ECABA total	18	18–90	35.5 ± 13.2	35 (24–44)	0.921	**0.924**
Physical consequences	5	5–25	11.0 ± 4.6	11 (7–15)	0.879	0.886
E-cig vs. packed cigarettes	5	5–25	10.7 ± 5.8	10 (5–14)	0.940	0.942
Establishing identification	3	3–15	5.2 ± 2.7	3 (3–7)	0.870	0.882
E-cigarette addiction	3	3–15	5.0 ± 2.2	5 (3–6)	0.752	0.805
Socialization	2	2–10	3.6 ± 1.8	3 (2–5)	0.501	0.525
EDS-22 (current users)	22	0–4	1.80 ± 1.18	2.00 (0.36–2.00)	0.985	**0.988**

ECABA, E-cigarette Attitude and Belief Scale in Adolescents; EDS-22, E-cigarette Dependence Scale; SD, standard deviation; IQR, interquartile range; α, Cronbach’s alpha; ω, McDonald’s omega. Higher scores indicate stronger pro–e-cigarette attitudes / greater dependence.

### 3.5 Attitudes and beliefs by use status

ECABA total and four of the five subscale scores increased significantly across non-users, occasional users, and regular users (*p* < 0.001 for the total, ε^2^ = 0.172; Table 5; Figure 2). For the total score, regular users differed from both non-users and occasional users, whereas non-users and occasional users did not differ (Table 5). The socialization subscale did not differ significantly across use groups (*p* = 0.064).

**Table 5:** ECABA total and subscale scores (medians) by current e-cigarette use.

Scale	Non-user (*n* = 302)	Occasional (*n* = 11)	Regular (*n* = 53)	*p*
ECABA total	33.0 a	39.0 a	48.0 b	**<0.001**
Physical consequences	10.0 a	13.0 a	15.0 b	**<0.001**
E-cig vs. packed	9.0 a	10.0 ab	15.0 b	**<0.001**
Establishing identification	3.0 a	4.0 a	9.0 b	**<0.001**
E-cigarette addiction	4.0 a	5.0 ab	6.0 b	**0.007**
Socialization	3.0	4.0	6.0	0.064

Values are medians. Kruskal–Wallis test; different superscript letters (a, b) indicate a significant pairwise difference (Dunn test, Bonferroni-adjusted). Bold p-values are significant. ECABA, E-cigarette Attitude and Belief Scale in Adolescents.

**Figure 2:**
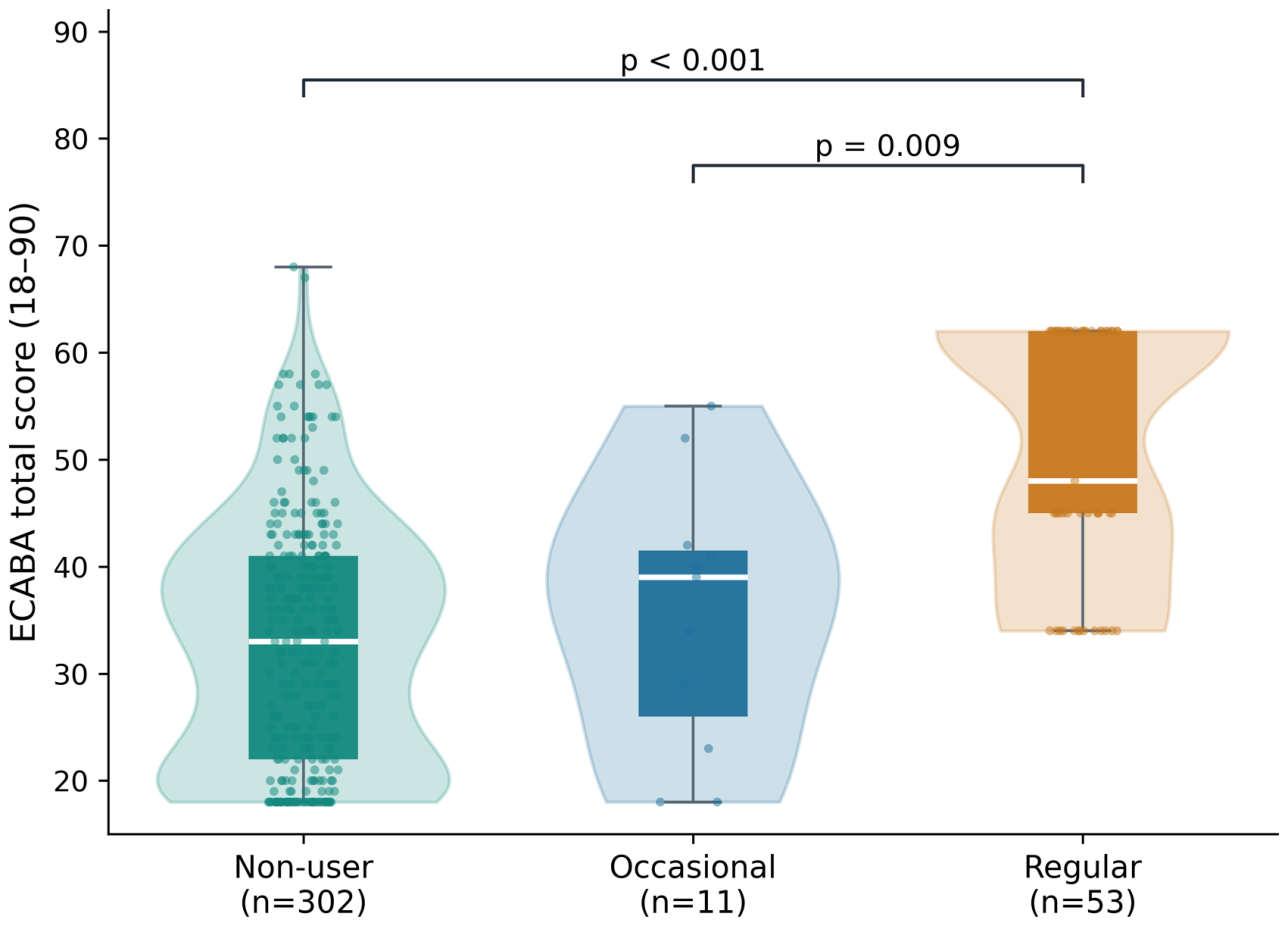
Distribution of ECABA total scores across non-users, occasional users, and regular users of e-cigarettes (higher scores indicate more pro–e-cigarette attitudes).

### 3.6 Dependence among current users

Among current users, EDS-22 dependence was higher in regular than occasional users (medians 2.00 vs. 0.45; *p* < 0.001, rank-biserial *r* = 0.636) and did not differ by sex (*p* = 0.660, rank-biserial *r* = 0.066) (Table 6). The ECABA total score correlated positively with EDS-22 dependence (*r* = 0.561, *p* < 0.001; Figure 3).

**Table 6:** E-cigarette dependence (EDS-22) among current users (*n* = 64).

Comparison	Group (*n*)	EDS-22 median (IQR)	*p*
Use frequency	Regular (*n* = 53)	2.00 (1.86–2.00)	**<0.001**
	Occasional (*n* = 11)	0.45 (0.05–1.80)	
Sex	Male (*n* = 43)	2.00 (0.36–2.00)	0.660
	Female (*n* = 21)	2.00 (0.36–2.00)	

Mann–Whitney U test. ECABA total correlated with EDS-22 dependence (Spearman *r* = 0.561, *p* < 0.001). Bold *p*-values are significant. EDS-22, E-cigarette Dependence Scale; IQR, interquartile range.

**Figure 3:**
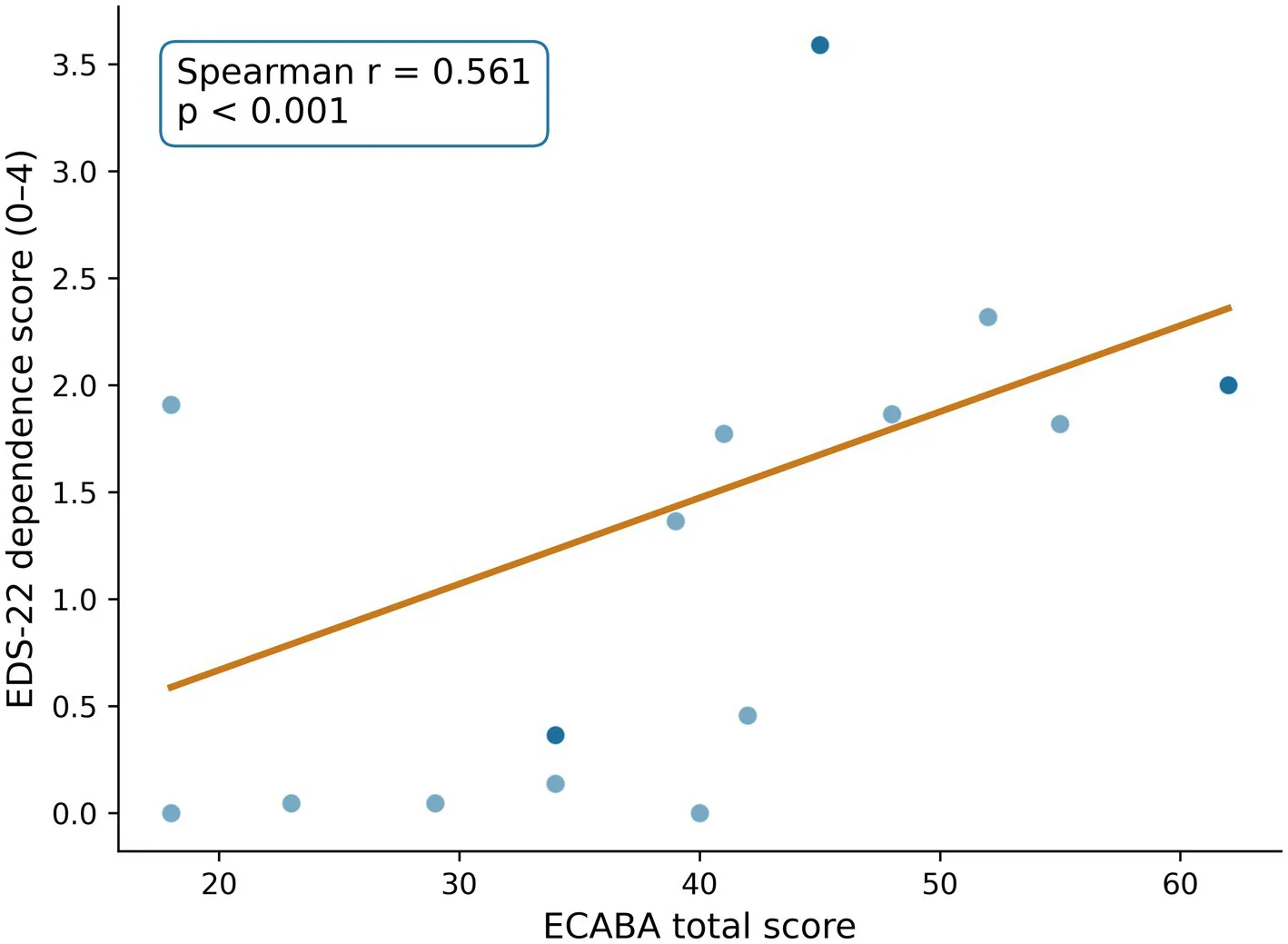
Correlation between ECABA total score (attitudes/beliefs) and EDS-22 dependence among current users (*n* = 64).

### 3.7 Predictors of current E-cigarette use

In the multivariable logistic regression model, male sex (aOR 2.45, 95% CI 1.27–4.75) and a higher ECABA total score (aOR 2.72 per 10 points, 95% CI 2.08–3.54) independently predicted current e-cigarette use, whereas age, current cigarette smoking, and household smoking did not (Table 7; Figure 4). The model showed adequate calibration (Hosmer–Lemeshow *p* = 0.276). No multicollinearity was detected among the predictors (all variance inflation factors ≤ 1.20).

**Table 7:** Multivariable logistic regression for current e-cigarette use (*n* = 365).

Predictor	aOR	95% CI	*p*
Male (vs female)	2.45	1.27–4.75	**0.008**
Age (per year)	1.02	0.90–1.15	0.768
Current cigarette smoking	1.83	0.89–3.75	0.101
Household smoker	0.98	0.49–1.99	0.965
ECABA total (per 10 points)	2.72	2.08–3.54	**<0.001**

aOR, adjusted odds ratio; CI, confidence interval. Nagelkerke R^2^ = 0.25; Hosmer–Lemeshow *p* = 0.276. Bold *p*-values are significant. No multicollinearity was detected (all VIF ≤ 1.20). VIF, variance inflation factor. One participant was excluded from the model owing to a missing age value.

**Figure 4:**
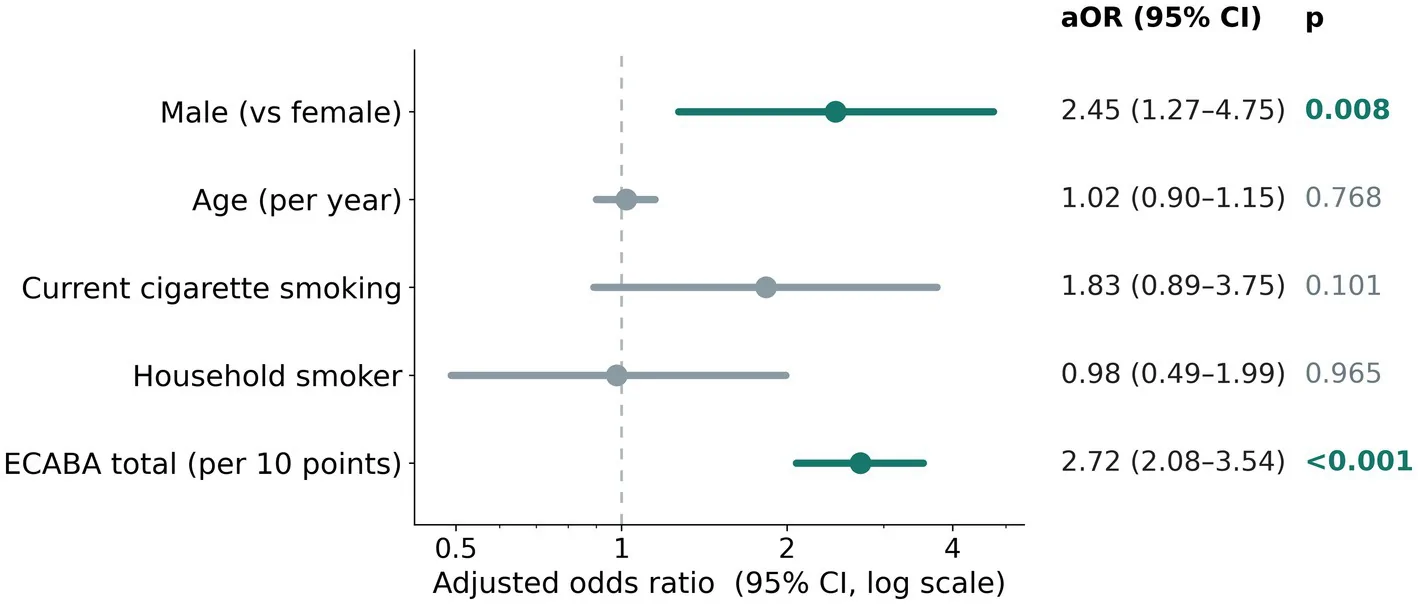
Adjusted odds ratios (95% CI) for current e-cigarette use from the multivariable logistic regression model (independent of age, cigarette smoking, and household exposure).

## 4 Discussion

In this cross-sectional study of 366 medical students, roughly one in three had experimented with e-cigarettes and 17.5% were current users. Pro–e-cigarette attitudes and beliefs, quantified with the ECABA, rose steadily from non-users to occasional to regular users and emerged as the strongest independent correlate of current use, exceeding conventional risk factors. Among current users, these attitudes were also closely tied to dependence.

The current-use prevalence observed here is broadly consistent with contemporary estimates. A global meta-analysis of students reported a pooled prevalence of 22.7% (2), while a large study of medical students in Türkiye documented a similar order of magnitude despite the national prohibition on e-cigarette sales (4). University-based studies from other settings have likewise reported use in the range of one in six to one in four (11, 12, 15). That a substantial minority of medical students—future sources of tobacco-cessation advice—are current users underscores the practical relevance of the problem. Comparable or higher figures have been reported from settings where e-cigarettes are legally sold: multinational surveillance spanning countries with legal retail markets documents substantial young-adult use (18), and university-based studies from such settings report prevalences in a similar range (11, 12), indicating that the prohibition context does not place our sample outside the international spectrum.

Male sex independently more than doubled the odds of current use, echoing the consistent male predominance reported in meta-analytic and multinational data (2, 18). Notably, however, attitudes and beliefs did not differ by sex, even though use did. This dissociation suggests that the sex gap in behaviour is not simply a reflection of a corresponding gap in cognitions and may instead be shaped by social, contextual, or opportunity-related factors.

The dominance of the ECABA score in the adjusted model is the study’s principal finding and aligns with long-standing behavioural theory. Both the Theory of Planned Behaviour (9) and the Health Belief Model (10) predict that favourable beliefs about a product’s risks and social value drive its uptake. The pattern within the subscales is instructive: differences between users and non-users were most pronounced for beliefs comparing e-cigarettes favourably with conventional cigarettes and for identity-related beliefs, mirroring reports that perceiving e-cigarettes as relatively harmless is a robust correlate of use (11). That current cigarette smoking lost significance once attitudes were accounted for reinforces the interpretation that beliefs are the proximal cognitive driver.

Among current users, dependence measured with the EDS-22 was substantial and scaled with use frequency, being markedly higher in regular than occasional users. The positive correlation between attitudes and dependence indicates that the same favourable cognitions associated with initiating and maintaining use are also linked to its escalation. This is clinically meaningful: dependence is the pathway through which experimentation becomes entrenched and through which the risk of transition to combustible tobacco is thought to operate (5–7).

For medical education specifically, these results carry a dual implication. First, the students who will soon counsel patients are themselves not immune to the attitudes that promote vaping, and their formal preparation on e-cigarettes is frequently inadequate (4, 14). Second, because the key correlate is a set of beliefs rather than a fixed demographic trait, it is amenable to educational change. From an education-science perspective, durable modification of health-related beliefs is best achieved through structured, competency-based curricula that combine accurate risk communication with the critical-appraisal and behaviour-change skills that underpin effective health education. Notably, current use was higher among preclinical than clinical students (21.1% vs. 11.5%), even though attitudes did not differ by phase, suggesting that the earlier years of training may be a particularly opportune window for preventive education, before use becomes established. In concrete terms, such content could comprise a dedicated module on novel nicotine products within existing tobacco teaching; training in brief-intervention frameworks (e.g., the 5A’s) adapted to vaping; critical appraisal of e-cigarette marketing claims; and assessment of these competencies in clinical-skills examinations.

Finally, the context of a national sales ban deserves comment. Despite prohibition, one in six students was a current user, and access was reported predominantly through friends and social media rather than formal retail. This indicates that supply-side prohibition alone does not eliminate availability and highlights the need to pair regulation with demand-side measures—education, counter-marketing, and denormalisation—that act on the attitudes and social channels through which these products actually reach young people (1, 19).

### 4.1 Limitations

Several limitations should be considered. First, the cross-sectional design precludes causal inference; the associations between attitudes, use, and dependence are correlational. Second, all measures were self-reported and may be subject to recall and social-desirability bias, although anonymity was assured. Third, the study was conducted at a single faculty, which may limit generalisability. Fourth, dependence was assessed only among current users (*n* = 64); the occasional-user subgroup was small (*n* = 11), limiting the power of pairwise comparisons, and the two-item socialization subscale showed low internal consistency (α = 0.501), so results for this subscale should be regarded as exploratory. Finally, the ECABA was originally validated in adolescents; although its items are content-appropriate for young adults and it showed excellent internal consistency here, its application to university students should be interpreted with this provenance in mind. In addition, although participants were selected by stratified random sampling, participation was voluntary, and students with a particular interest in—or experience of—e-cigarettes may have been more inclined to respond, introducing potential self-selection bias; the high response rate (88.1%) mitigates but does not eliminate this concern. Furthermore, despite multivariable adjustment, residual confounding by unmeasured factors (e.g., peer networks, other substance use, or psychological traits) cannot be excluded.

## 5 Conclusion

Among medical students at an eastern Anatolian faculty, e-cigarette experimentation and current use were common, and pro–e-cigarette attitudes and beliefs were the strongest independent correlate of both use and, among users, dependence. Because these cognitions are modifiable, embedding e-cigarette-specific, competency-based content within medical and health-education curricula—alongside demand-side public-health measures—may offer a promising avenue for reducing use in this group; given the cross-sectional design, however, causal inferences should be drawn cautiously, and the impact of such curricular interventions should be evaluated prospectively.

## Data Availability

The raw data supporting the conclusions of this article will be made available by the authors, without undue reservation.

## References

[1] Golder S Hartwell G Barnett LM Nash SG Petticrew M Glover RE. Vaping and harm in young people: an umbrella review. Tob Control. (2025). doi: 10.1136/tc-2024-059219 40829950

[2] Gebeyehu NA Gelaw KA Atalay YA Adella GA. Global prevalence of e-cigarette use among students: systematic review and meta-analysis. PLoS One. (2025) 20:e0332160. doi: 10.1371/journal.pone.0332160 PMC12668485 41325320

[3] Kurtuluş Ş Can R. Use of e-cigarettes and tobacco products among youth in Turkey. Eurasian J Med. (2022) 54:127–32. doi: 10.5152/eurasianjmed.2022.20168, 35703519 PMC9634893

[4] Dilektaşli AG Güçlü OA Özpehlivan A Coşkun F Ursavaş A Karadağ M . Electronic cigarette use and consumption patterns in medical university students. Front Public Health. (2024) 12:1403737. doi: 10.3389/fpubh.2024.1403737 PMC11472834 39403440

[5] Chan GCK Stjepanović D Lim C Sun T Shanmuga Anandan A Connor JP . Gateway or common liability? A systematic review and meta-analysis of adolescent e-cigarette use and future smoking initiation. Addiction. (2021) 116:743–56. doi: 10.1111/add.15246 32888234

[6] Begh R Conner M Lindson N Fanshawe TR Cheah S Hartmann-Boyce J. Electronic cigarettes and subsequent cigarette smoking in young people: a systematic review. Addiction. (2025) 120:1090-1111. doi: 10.1111/add.16773 PMC12046492 39888213

[7] Kandel ER Kandel DB. A molecular basis for nicotine as a gateway drug. N Engl J Med. (2014) 371:932–43. doi: 10.1056/NEJMsa1405092, 25184865 PMC4353486

[8] Hajek P Etter JF Benowitz N Eissenberg T McRobbie H. Electronic cigarettes: review of use, content, safety, effects on smokers and potential for harm and benefit. Addiction. (2014) 109:1801–10. doi: 10.1111/add.12659, 25078252 PMC4487785

[9] Ajzen I. The theory of planned behavior. Organ Behav Hum Decis Process. (1991) 50:179–211. doi: 10.1016/0749-5978(91)90020-T

[10] Rosenstock IM. Historical origins of the health belief model. Health Educ Monogr. (1974) 2:328–35. doi: 10.1177/109019817400200403, 42475469

[11] Kurdi R Al-Jayyousi GF Yaseen M Ali A Mosleh N Abdul Rahim HF. Prevalence, risk factors, harm perception, and attitudes toward e-cigarette use among university students in Qatar: a cross-sectional study. Front Public Health. (2021) 9:682355. doi: 10.3389/fpubh.2021.682355, 34490180 PMC8417713

[12] Nazzal Z Maraqa B Azizeh R Odeh R. Exploring the prevalence, knowledge, attitudes and influencing factors of e-cigarette use among university students in Palestine: a cross-sectional study. BMJ Open. (2024) 14:e080881. doi: 10.1136/bmjopen-2023-080881 PMC10875484 38367977

[13] Oksay SC Alpar G Bilgin G Çakır E Girit S. E-cigarette attitude and belief scale in adolescents: a validity and reliability study. Thorac Res Pract. (2025) 26:290–7. doi: 10.4274/ThoracResPract.2025.2025-5-5 PMC12572912 40960387

[14] Mali S Chiodze V Jersmann H. Clearing the fog: Australian medical students and the e-cigarette knowledge void—a cross-sectional survey. BMC Med Educ. (2025) 25:1612. doi: 10.1186/s12909-025-08126-2, 41250059 PMC12625367

[15] Ghanim M Rabayaa M Abuawad M Saeedi M Amer J. E-cigarette use among university students in Palestine: prevalence, knowledge, and determinant factors. PLoS One. (2024) 19:e0302946. doi: 10.1371/journal.pone.0302946, 38718008 PMC11078419

[16] Morean ME Krishnan-Sarin S Sussman S Foulds J Fishbein H Grana R . Psychometric evaluation of the e-cigarette dependence scale. Nicotine Tob Res. (2019) 21:1556–64. doi: 10.1093/ntr/ntx271, 29301008 PMC6821314

[17] Aslan B Onal O. Validity and reliability of four instruments for e-cigarette dependence and sensory factors in e-cigarette use. J Drug Issues. (2026) 56:17–34. doi: 10.1177/00220426241265629

[18] Pan L Morton J Mbulo L Dean A Ahluwalia IB. Electronic cigarette use among adults in 14 countries: a cross-sectional study. EClinicalMedicine. (2022) 47:101401. doi: 10.1016/j.eclinm.2022.101401, 35497060 PMC9046112

[19] Glantz SA Bareham DW. E-cigarettes: use, effects on smoking, risks, and policy implications. Annu Rev Public Health. (2018) 39:215–35. doi: 10.1146/annurev-publhealth-040617-013757, 29323609 PMC6251310

