# Supplementary material for: Attitudes, beliefs, and dependence related to electronic cigarettes among medical students at an eastern Anatolian university: a cross-sectional study
Source: Front Public Health. 2026 Sep 24;14:1934215. doi: 10.3389/fpubh.2026.1934215 (PMC13646052; doi:10.3389/fpubh.2026.1934215)
Supplement: Supplementary file 1 [file Supplementary_file_1.docx]

**Supplementary Materials**

*Attitudes, Beliefs, and Dependence Related to Electronic Cigarettes Among Medical Students at an Eastern Anatolian University: A Cross-Sectional Study*

**Table S1.** Extended sociodemographic characteristics of the study sample (N = 366).

| **Characteristic** | **Category** | **n (%)** |
| --- | --- | --- |
| Marital status | Single | 351 (95.9) |
|  | Married | 15 (4.1) |
| Living arrangement | With family | 164 (44.8) |
|  | Dormitory | 148 (40.4) |
|  | Student house | 33 (9.0) |
|  | Alone | 21 (5.7) |
| Longest place of residence | Metropolitan city | 173 (47.3) |
|  | Provincial city | 111 (30.3) |
|  | District town | 63 (17.2) |
|  | Village | 19 (5.2) |
| Family structure | Nuclear family | 314 (85.8) |
|  | Extended family | 43 (11.7) |
|  | Single-parent / disrupted | 9 (2.5) |

*Values are n (%). Percentages are of the total sample (N = 366).*

**Table S2.** Spearman correlation matrix among ECABA subscales and total score (N = 366).

| **Subscale** | **1** | **2** | **3** | **4** | **5** | **Total** |
| --- | --- | --- | --- | --- | --- | --- |
| 1. Physical consequences | 1.000 | 0.683 | 0.419 | 0.566 | 0.203 | 0.850 |
| 2. E-cig vs packed cigarettes | 0.683 | 1.000 | 0.420 | 0.719 | 0.208 | 0.878 |
| 3. Establishing identification | 0.419 | 0.420 | 1.000 | 0.433 | 0.416 | 0.664 |
| 4. E-cigarette addiction | 0.566 | 0.719 | 0.433 | 1.000 | 0.341 | 0.790 |
| 5. Socialization | 0.203 | 0.208 | 0.416 | 0.341 | 1.000 | 0.420 |
| ECABA total | 0.850 | 0.878 | 0.664 | 0.790 | 0.420 | 1.000 |

*All correlations were statistically significant (p < 0.001). ECABA, E-cigarette Attitude and Belief Scale in Adolescents.*

**Table S3.** Crude and adjusted odds ratios for current e-cigarette use (n = 365).

| **Predictor** | **Crude OR (95% CI)** | **p** | **Adjusted OR (95% CI)** | **p** |
| --- | --- | --- | --- | --- |
| Male (vs female) | 2.26 (1.28–4.00) | **0.005** | 2.45 (1.27–4.75) | **0.008** |
| Age (per year) | 1.05 (0.95–1.16) | 0.364 | 1.02 (0.90–1.15) | 0.768 |
| Current cigarette smoking | 1.95 (1.12–3.40) | **0.019** | 1.83 (0.89–3.75) | 0.101 |
| Household smoker | 1.53 (0.87–2.69) | 0.138 | 0.98 (0.49–1.99) | 0.965 |
| ECABA total (per 10 points) | 2.65 (2.05–3.43) | **<0.001** | 2.72 (2.08–3.54) | **<0.001** |

*OR, odds ratio; CI, confidence interval. The adjusted model included all listed predictors simultaneously (Nagelkerke R² = 0.25; Hosmer–Lemeshow p = 0.276). Statistically significant p-values are shown in bold.*

**Table S4.** Collinearity diagnostics for the multivariable logistic regression model.

| **Predictor** | **VIF** | **Tolerance** |
| --- | --- | --- |
| Male sex | 1.05 | 0.952 |
| Age | 1.06 | 0.943 |
| Current cigarette smoking | 1.20 | 0.833 |
| Household smoker | 1.11 | 0.901 |
| ECABA total (per 10 points) | 1.01 | 0.990 |

*VIF, variance inflation factor; tolerance = 1/VIF. All VIF values ≤ 1.20 indicate the absence of multicollinearity. ECABA, E-cigarette Attitude and Belief Scale in Adolescents.*
