# Supplementary material for: Attitudes, beliefs, and dependence related to electronic cigarettes among medical students at an eastern Anatolian university: a cross-sectional study
Source: Front Public Health. 2026 Sep 24;14:1934215. doi: 10.3389/fpubh.2026.1934215 (PMC13646052; doi:10.3389/fpubh.2026.1934215)
Supplement: Supplementary file 2 [file Supplementary_file_2.docx]

**STROBE Statement — Checklist for Cross-Sectional Studies**

*Attitudes, Beliefs, and Dependence Related to Electronic Cigarettes Among Medical Students*

| **Item** | **Recommendation (STROBE, cross-sectional)** | **Reported / location in manuscript** |
| --- | --- | --- |
| 1 | Title and abstract — study design in title; structured summary | Title states 'cross-sectional study'; structured abstract (Background/Methods/Results/Conclusions). |
| 2 | Introduction — background/rationale | Introduction, paragraphs 1–4. |
| 3 | Objectives | Introduction, final paragraph (aims i–iv). |
| 4 | Study design | Methods, Section 2.1 (cross-sectional; STROBE-compliant). |
| 5 | Setting, locations, dates | Methods, Section 2.1 (İnönü University Faculty of Medicine; 2025–2026). |
| 6 | Participants — eligibility, sources, selection | Methods, Section 2.2; Figure 1 (term-stratified random sampling). |
| 7 | Variables — outcomes, exposures, predictors | Methods, Sections 2.3–2.4 (ECABA, EDS-22, current use; model predictors). |
| 8 | Data sources / measurement | Methods, Section 2.3 (validated ECABA and EDS-22 instruments). |
| 9 | Bias | Discussion, Section 4.1 (self-report/social-desirability; anonymity). |
| 10 | Study size | Methods, Section 2.2 (OpenEpi; minimum 318 + 15%; target 366). |
| 11 | Quantitative variables — handling | Methods, Sections 2.3–2.4 (scale scoring; ECABA modelled per 10 points). |
| 12 | Statistical methods | Methods, Section 2.4 (SPSS 22; non-parametric tests; logistic regression; Hosmer–Lemeshow). |
| 13 | Participants — numbers at each stage | Results, Section 3; Figure 1 (379 received → 13 excluded → 366 analysed). |
| 14 | Descriptive data | Results, Sections 3.1–3.2; Tables 1–2. |
| 15 | Outcome data | Results, Sections 3.2–3.5; Tables 2–5. |
| 16 | Main results — estimates, CIs, adjustment | Results, Section 3.7; Table 7; Figure 4 (adjusted ORs with 95% CI). |
| 17 | Other analyses — subgroups, interactions | Results, Sections 3.3–3.6; Tables 3–6 (by educational phase, use group, sex, frequency). |
| 18 | Key results — summary with reference to objectives | Discussion, paragraph 1. |
| 19 | Limitations | Discussion, Section 4.1. |
| 20 | Interpretation | Discussion, paragraphs 2–7. |
| 21 | Generalisability | Discussion, Section 4.1 (single-centre). |
| 22 | Funding | Declarations (Funding: none). |

*Locations refer to the section headings, tables, and figures of the manuscript. STROBE: Strengthening the Reporting of Observational Studies in Epidemiology.*
